# Differential Experiences of Intimate Partner Violence during COVID-19: A Cross-Regional Study in Mexico

**DOI:** 10.3390/bs14040331

**Published:** 2024-04-15

**Authors:** Sofia Navarrete Zur, Paola M. Sesia

**Affiliations:** 1 COMEXUS Fulbright Research Scholarship Program at the Center for Research and Advanced Studies in Social Anthropology (CIESAS) Pacífico-Sur, Oaxaca 68024, Mexico; 2 CIESAS Pacífico-Sur, Oaxaca 68024, Mexico; sesia@ciesas.edu.mx

**Keywords:** intimate partner violence, gender-based violence, COVID-19, urban–rural divide, socio-economic factors, stressors qualitative method

## Abstract

The COVID-19 pandemic brought on a marked increase in intimate partner violence (IPV) worldwide, Mexico being no exception. Factors that exacerbated gender-based violence (GBV) in the household during the pandemic include gendered loss of income, regression in access to social and legal justice resources, reversal to more traditional gender norms and roles, and increased alcoholism. While there are studies about the prevalence and determinants of IPV in rural and urban Mexico, there appears to be a lack of information regarding how these realities differed as they interacted with the compounding pressures of the COVID-19 pandemic. Stemming from 10 ethnographic interviews with women across rural and urban localities of Oaxaca, Mexico City, and Mexico State, who were recruited from NGOs providing psychological and legal services against GBV, we analyze some factors associated with the prevalence of IPV during confinement. We conclude that all women in our study experienced IPV both before and during the pandemic, with variations in IPV patterns influenced by their rural or urban residence, socio-economic status, ethnic-racial identity, and proximity to the abuser’s network. We also found that not all impacts were negative, rather COVID-19 measures had a paradoxical effect for some women where restrictions on geographical mobility and decrease in access to alcohol became pivotal protective factors. We recommend that public policymakers and civil society organizations alike pay attention to these differential challenges and benefits in their crisis responses.

## 1. Introduction

The COVID-19 pandemic brought about an immediate halt to global economic and social activities. Governments implemented stay-at-home orders, closed non-essential businesses, restricted travel, and discouraged social gatherings. This backdrop provided a context for examining the relationship between the pandemic and intimate partner violence (IPV). The United Nations General Secretary has labeled violence against women a “global pandemic” [1]. In 2020 nearly 47,000 women and girls were killed by their partners or other family members [2]. A substantial body of research has since explored the intersection of COVID-19 with increased IPV risk factors and severity worldwide [2,3,4,5,6,7,8,9,10]. Concurrently, UN Women reports that during the pandemic, at least 243 million women and girls aged 15 to 49 experienced IPV [9]. These studies highlight how crises, such as pandemics, exacerbate gender inequalities and hinder women’s access to healthcare, social services, and support, providing a broader context to understand the surge in IPV during this period.

In Mexico, research reveals a notable increase in 911 calls for IPV, a rise in women seeking shelters, and an uptick in femicides [5,6,8,10]. Responding to the pandemic’s strain on the legal system [11], marked by court closures and delays, Mexico improved online access to emergency services. Despite these initiatives, only 11% of women assaulted during the pandemic reached out to authorities, encountering either indifference or insufficient responses [12].

Studies have examined IPV in rural versus urban settings, highlighting how pre-existing discrimination, marital status [13], institutional racism, socio-economic and ethnic inequalities [14], and limited service access worsen IPV in rural areas in Mexico and beyond [10,15]. Most of these studies pre-date the COVID-19 pandemic [13,16], therefore there is a need for more focused and nuanced inquiries into the different experiences of IPV during this period. 

Our study examines the impact of the COVID-19 pandemic on IPV in Mexico through 10 case studies of women from different socio-economic and geographical contexts aiming to analyze women’s diverse experiences. We begin by discussing the prevalence and nature of gender-based violence (GBV) in Mexico, setting the groundwork for a discussion on the country’s localized responses to the pandemic. We then examine how these responses have affected IPV dynamics, focusing on the narratives of the 10 women. First, we point out that IPV was already present in their lives prior to the onset of the COVID-19 pandemic. Then, we delve into the multifaceted impacts of the pandemic and associated public health measures on women, with a particular focus on how these effects varied across different socio-economic strata, geographic regions, and within the context of women’s proximity to their abusers’ networks. Lastly, we explore the complex and sometimes contradictory consequences of COVID-19 interventions on IPV, highlighting their implications for women’s safety and well-being. This section aims to illuminate the shortcomings in existing support structures and escape mechanisms for women, which were starkly exposed during the pandemic.

## 2. Background

### 2.1. GBV & IPV in Mexico

In Mexico, violence against women pervades all areas of society [10]. According to the National Survey carried out every 5 years in Mexico [17], in 2021 43% of women were victims of GBV. Furthermore, a considerable number of femicides are perpetuated in family, couple, or friendship environments [17], with numbers rising [18]. In this study, we adopt the definition of IPV as outlined by the ENDIREH, 2021: actions of power abuse or intentional neglect aimed at dominating, subjugating, controlling, and/or assaulting women [17]. These actions encompass physical, verbal, psychological, vicarious, economic, or sexual violence, within and/or outside the home setting. The abuser is considered an individual who is or was in a marital or cohabitation relationship with the victim, or who has been in a similar intimate partnership. This definition includes “vicarious violence”, which involves using children as tools of coercion to psychologically harm the mother [19].

According to a 2022 World Health Organization report [20], 26% of women worldwide have experienced IPV by a current or former male partner, with this figure rising to 30% in the Americas. The ENDIREH survey across Mexico, however, reports a markedly higher rate, with 70% of women reporting exposure to one or more types of violence, a 4% increase from 2016. Within these statistics, 40% of violence occurred in a current or past relationship [17]. These alarming figures align with rising crime rates against women in Mexico since the onset of the Drug War in 2007 [21], underscored by an Amnesty International report that 11 women are killed daily in Mexico [22].

The three sites examined in this study—the state of Oaxaca, Mexico City, and the State of Mexico—exhibit high rates of violence against women: 67%, 76%, and 79%, respectively, with Mexico City and the State of Mexico having the highest rates nationwide [23]. These figures highlight a broader issue of GPV and IPV being deeply entrenched in societal norms, often concealed by stigma within families, social circles, and communities. This stigma contributes to widespread silence among victims. Secondary victimization, involving further trauma from healthcare, the justice system, and personal relationships [24], complicates matters and may contribute to the underreporting of IPV due to inadequate support discouraging incident reporting [25].

### 2.2. The COVID-19 Pandemic & Mexico’s Response

Mexico faced the COVID-19 pandemic in March 2020, aligning with the WHO’s declaration on 11 March [20]. The response included school closures [26], restricted public movement, and recommended stay-at-home guidelines, avoiding a national lockdown but limiting activities for schools, restaurants, and businesses until May’s end [27]. In June 2020, states adopted a traffic-light system, determined by new COVID-19 case counts, to gradually reopen businesses and public services. This aimed to mitigate the economic and social effects of prior restrictions. Therefore, the national response was not uniform, with variations in state responses set against a backdrop of pre-existing territorial and social inequalities in health service coverage and quality.

During the COVID-19 pandemic, Mexico refrained from introducing new safety nets or direct income support measures for households [24]. Instead, the federal government opted for alternative assistance methods such as offering credit to small and medium-sized enterprises and authorized banks to defer payments for months [5].

Mexico’s pandemic management strategy was characterized by decentralization, leading to varied local responses. This variation was not just between states, but also among municipalities, resulting in differing regional approaches to the pandemic. In urban areas, responses generally aligned with federal guidelines, while rural communities saw more drastic measures from local leaders. This included erecting barriers at community entrances and establishing local civil guards to control access [15]. Some areas imposed alcohol bans or restrictions [28].

A notable example of a local response was observed in the state of Oaxaca, where many municipalities operate under a legally recognized form of self-government that allows for a high degree of autonomy and is rooted in indigenous traditions and practices [29]. During the first year of the pandemic, community assemblies in these areas opted for exceptionally strict confinement measures. They isolated communities and enforced severe limitations on movement, both into and out of these areas, and sometimes even within the towns themselves. Additionally, there was a widespread restriction on alcohol consumption.

### 2.3. IPV during the COVID-19 Pandemic 

During the pandemic, Mexico’s austerity-driven approach led to prioritizing COVID-19 hospitals in response to the public health emergency. The national response lacked a focused strategy to address the potential risks associated with the recommended practices of confinement at home. This gap in policy was particularly critical given the nature of domestic violence (DV) as the most prevalent form of violence against women in Mexico [10]. The pandemic amplified factors like stress and economic uncertainty which are known to trigger abuse, while confinement cuts off women from their support networks, increasing their vulnerability [10]. Furthermore, confinement highlighted and intensified existing gender inequalities in unpaid labor distribution [23]. The lack of a comprehensive strategy or adequate state support left significant gaps in protecting women’s and children’s well-being and safety, while underscoring deep-seated gender inequalities in the home.

The ENDIREH report highlights varied pandemic effects on IPV rates, with state-specific differences. Oaxaca saw 39% of women experiencing violence, below the national average of 43%. In contrast, Mexico City reported a higher rate of 46%, and the State of Mexico nearly 48% [17]. Mexican government data indicate a significant rise in GBV during the early stages of the COVID-19 pandemic, with a 22% increase in femicides in March–April 2020 compared to the same period in the previous year [30]. The government’s passive stance to escalating GBV and IPV during the pandemic was alarming, with Alvarez-Piñeiro [30] highlighting the lack of prioritization given to femicides within the national agenda and the absence of clear policies to combat the surge in IPV and GBV. In a country where violence against women is systemic, activist groups have emerged as the primary safety network for many women [6]. Their role becomes increasingly crucial in the face of governmental apathy and the perpetuation of gender stereotypes and inequalities, further highlighting the gap between the severity of the issue and the official response.

## 3. Materials and Methods

This research employed a longitudinal qualitative approach, spanning 9 months from October 2022 to July 2023. It incorporated extensive semi-structured ethnographic interviews complemented by participant observation allowing for an in-depth understanding of each individual’s experiences over an extended period. The study was conducted in diverse environments, ranging from rural locales to urban centers within the State of Oaxaca, the State of Mexico, and Mexico City. 

Interviews were conducted by the PI in collaboration with local NGOs dedicated to supporting gender-based violence survivors. Partnerships included Grupo de Estudios sobre la Mujer Rosario Castellanos A.C. (GES Mujer) in Oaxaca, FemXFem in the State of Mexico, and Equis Justicia para las Mujeres in Mexico City. The PI volunteered with these organizations, offering research consultation services, Spanish to English translation support whenever needed for their projects and grants, and logistical support during workshops in remote indigenous areas of Oaxaca. This commitment granted access to spaces and events where women could learn about the study and decide whether they wanted to participate. This approach fostered mutual benefits for both the organizations and the research. Women’s participation in the study was based on their own choice and willingness to engage.

Eligibility criteria for participants required them to be over 18 years old and Spanish-speaking; all selected participants had experienced IPV during the pandemic. All participants were fully briefed about the study’s objectives and procedures and provided written informed consent prior to participation. All participants are referenced using pseudonyms to ensure confidentiality and protect their identities. The institutional review board of The New School Graduate School approved the research. 

### 3.1. Participants

The research involved 10 women who were interviewed extensively during a period of several months in a safe environment of their choosing, usually at their homes or refuge. In Oaxaca, six women were interviewed, representing a spectrum of identities including indigenous and mestiza women, and socio-economic backgrounds ranging from low to middle class. Three of the localities are indigenous and far removed from the state capital, whereas two others are located in the outskirts of the city of Oaxaca and are inhabited by local and migrant populations with diverse ethnic backgrounds. In the State of Mexico, two interviews were conducted with women who belong to the upper socio-economic class and reside in urban peripheries. Both women identify themselves ethnically as mestizas. Additionally, two participants from Mexico City, coming from middle- to upper-class backgrounds, also identified as mestizas. Significantly, one participant has dual nationality, Mexican and U.S. citizenship. Their main socio-demographic and socio-economic characteristics, including their ethnicity and residence status, are summarized in the following Table 1:

Participants engaged in detailed discussions on their experiences as victims of IPV during the pandemic, including COVID-19-related confinements and the distress they felt during this period. During interviews, a clear pattern emerged where all women conveyed experiences of IPV prior to the pandemic; thus, we extended the interview to include the description of pre-pandemic experiences. Additionally, they reported their strategies of resistance, resilience, and healing during and after IPV encounters. Before delving into these topics, at the outset of the interviews, we collected demographic information including age, gender, race, education, income, and relationship status. 

We ensured a trauma-aware, participant-driven dialogue that was both engaging and sensitive to participants’ experiences. The interviews also included open-ended and follow-up questions to encourage a natural conversation flow. They were also flexible in time, which allowed for the discussions to evolve organically, empowering participants to guide the conversation including the pacing, the topics covered, the depth of their responses, and their level of anonymity. Some participants shared personal items such as photo albums and legal documents, providing deeper insights into their individual stories, social connections, and legal backgrounds. After the interviews, a brief debriefing session was carried out to discuss any feelings or reactions the participant might have experienced, reinforcing their value and the importance of their contribution. Follow-ups were also conducted to offer additional support if needed.

### 3.2. Data Processing and Analysis

All interviews were recorded with authorization by the participants. The data were stored under password-protected hard drives and were only accessed by the researchers. The interviews were fully transcribed, codified, and analyzed using ATLAS.ti Version 23.3.4. Names were erased and identifying details were changed for the protection of the participants. Systematic interview coding and subsequent code-document analysis were conducted to determine the frequency of recurring ideas, topics, or concepts across the dataset. The organization of codes into broader themes informed by grounded theory resulted in the identification of key factors associated with IPV during the pandemic-induced confinement measures and the strategies women employed to cope or seek support. Narrative analysis was employed to reveal how cultural norms, education levels, socio-economic situations, and available resources impacted women’s experiences and responses to violence during the pandemic in these diverse contexts. Lastly, we cross-referenced data from the ethnographic interviews and participant observations to help validate the emerging themes related to the factors associated with IPV and resilience mechanisms. This approach provided a comprehensive understanding of how these realities differed across different settings in the context of the COVID-19 pandemic.

## 4. Theoretical Framework 

### Grounded Theory

Grounded theory proved to be instrumental in navigating the complex and multifaceted nature of IPV during the pandemic. This theory, developed by Glaser and Strauss in 1967, is an inductive and flexible approach that is suited for exploring social phenomena that are both dynamic and contextual, such as IPV. It diverges from hypothesis-driven approaches, and enables the exploration of emergent themes, a feature that proved essential in studying the rapidly evolving and unprecedented context of the COVID-19 pandemic and the nuances and variations in IPV across different socio-demographic groups.

However, this approach requires a high degree of reflexivity due to its reliance on the researcher’s ability to interpret data, potentially introducing subjectivity into the analysis and theory development process. We were, therefore, especially vigilant in critically examining our biases, perspectives, and experiences to minimize their influence on data interpretation. Additionally, the lack of standardized procedures can lead to inconsistencies in the application of grounded theory across studies, complicating the comparison of results. Moreover, grounded theory’s dependence on the unique contexts of the data and the subjective interpretation by researchers poses challenges to replicating studies such as ours, which may hinder efforts to validate the findings generated from our research.

Notwithstanding these challenges, grounded theory is useful in anthropological and social research, especially in studies addressing complex social and cultural phenomena. Anthropologists such as Charmaz [31,32] and Clarke, alongside Friese and Washburn [33], have used grounded theory to investigate a wide range of social experiences. Specifically, Charmaz’s constructivist interpretation of grounded theory emphasizes the importance of understanding participants’ perspectives and constructing theories that are deeply rooted in their experiences [34]. This approach was particularly pertinent to our study, enabling an understanding of IPV during the COVID-19 pandemic that is grounded in the lived experiences of diverse women in Mexico. Clarke’s expansion of grounded theory into situational analysis provided a valuable framework for dissecting the complex interplay of personal, social, and environmental factors influencing IPV during the unique context of the pandemic [35].

We compared our results with existing literature, facilitating the identification of key themes and patterns, and enabling us to encapsulate the diverse, complex, and sometimes paradoxical experiences of IPV. These experiences are shaped by factors such as urban versus rural settings, socio-economic status, and ethnic backgrounds, which were crucial for comprehending the multifaceted nature of IPV during the pandemic.

## 5. Results

### 5.1. IPV before the Pandemic 

Before the COVID-19 pandemic, all participants had experienced IPV, as exemplified by Isabel, a 35-year-old indigenous mother living in economic hardship. Isabel first encountered IPV in 2016 while adhering to her role in managing household duties and caring for her children in her partner’s isolated rural community. She faced the “apex” of violence in her life with an attempted femicide in 2017 by the same partner, who continued his abuse during the pandemic.

*“He checked my phone and found texts with someone else. Though they weren’t compromising, he was drunk and erupted in anger telling me he was going to kill me. He beat me, stripped and dragged me. I escaped, screaming for help, but no one came. He continued, pressing his foot against my stomach and neck. He threatened me with a shotgun, first at my heart, then at my genitals. I prayed aloud. He stopped, threw me on the bed, and forced himself on me. I was in a critical condition for months after that. Even after everything, I still could not get out of that violent situation…it took me years from the last time he hit me to the time we separated, which was during the pandemic.”*

Like Isabel and many women in similar socio-economic and rural settings, traditional gender roles, social control, and passive community complicity underlined their experiences of prolonged abuse, extending into the pandemic. However, IPV transcended socio-economic and geographical boundaries, affecting middle- and upper-class urban women too. For instance, Julia, a 41-year-old mother from an urban, wealthier background, also faced IPV. Her husband, a renowned doctor, primarily inflicted psychological and economic harm through gaslighting, leading Julia to doubt her perceptions of his infidelity and financial exploitation.

*“He would buy cars and go on vacations with other women, using my credit and plunging me into debt. When I confronted him about infidelity and finances, he twisted the argument so convincingly that it caused me to question my reality and sanity.”*

This manipulative behavior led Julia to extreme debt and depression. The severity of her financial crisis and evident unhappiness eventually drew the attention of her family and friends, prompting them to intervene.

The patterns of violence in Julia’s experience echo those of women like Isabel, beginning with initial manipulative affection and escalating in severity. However, the type of violence varied. Isabel endured physical, psychological, and economic abuse, with her financial dependency used as a control mechanism. In contrast, upper-class women like Julia faced less physical but significant economic exploitation and psychological manipulation, with abuse tactics tailored to exploit their financial independence and wealth. Economic abuse also manifested differently: women in lower socio-economic positions suffered from direct financial withholding, while women like Julia experienced complex economic extortion, including manipulation for financial gain. The visibility of the abuse also varied; Isabel’s abuser was indifferent to public perception, whereas Julia’s abuser maintained a façade to protect his reputation, keeping the abuse private. Moreover, the support networks available to these women differed significantly. Isabel contended with societal pressure and isolation, while Julia received support and validation from her social circle, highlighting the varied experiences of IPV across different socio-economic backgrounds.

### 5.2. The Negative Influence of the Pandemic and COVID-Induced Measures

Distinct patterns of IPV and the varied responses from women’s support networks significantly influenced their experiences during the COVID-19 pandemic, affecting both how IPV manifested and was addressed in this timeframe. In particular, we found specificities in women’s IPV experiences related to socio-economic status, rural vs. urban residence, and the proximity or distance to abusers’ family and social networks.

#### 5.2.1. Influence of Socio-Economic Status and the Rural–Urban Divide 

Rita, a 42-year-old indigenous woman residing in a lower-class neighborhood in Oaxaca, works as a domestic worker, and is a single mother to one son. She explained to us how difficult it was for her to live on a precarious income of $1000 pesos weekly (an equivalent of ~$59 USD) in stable employment circumstances, and even more so during periods of irregular work. She shared her modest living space with the landlord and two other families. Her aggressor, a former partner and neighbor, resided nearby with his own family. 

The advent of the pandemic exacerbated Rita’s vulnerabilities. She lost her employment without any financial compensation, a common yet unlawful practice reflecting the widespread mistreatment of domestic workers in Mexico. 

Rita’s situation left her struggling to provide for herself and her son. Lockdown measures in her rural hometown prevented her return, further narrowing her options. She relied on the support of her social network for food and precarious odd jobs, which were inadequate for survival. Amid these challenging conditions, and increased confinement in the communal home, Rita faced escalating IPV from her ex-partner, including multiple sexual harassment incidents following a surgery she underwent early in the pandemic. Her constrained living situation made it impossible to extricate herself. Rita recounted a specific incidence of sexual assault:

*“I left my door ajar and he got into my room. I had just had a tumor removed. When I woke up, I felt that he was touching me. I said what are you doing? I don’t know where I got the strength from, but I pushed him and told him to get out or I would call the police. I got out of the room as soon as I could… The next day we thought about leaving, and I complained to the landlord, who said he’d deal with it, but he didn’t... We had nowhere to go. I had to face him there every day.”*

When Rita sought help, she could not access support services. Too scared to venture out of her neighborhood, she felt utterly abandoned by the state and bereft of viable coping mechanisms. “*They didn’t give us a penny. The government doesn’t care about us, and even less so if you’re a house worker like me*”. Her interactions with public institutions such as the prosecutor’s office in Judicial City, which were only partially operational during the confinement period, added to her growing disillusionment. These agencies provided limited assistance that lacked any substantial aid or resources.

*“First, I tried going [to the prosecutor’s office] many times and the front guard would turn me away, saying they couldn’t assist me. Then, when I was finally let in, they didn’t listen to me…They said, ‘Sorry, we can’t do anything. You haven’t been raped and you are not bleeding, so nothing happened to you. You’re fine.’ But I wasn’t fine… I left feeling really bad. They… looked down on me. They had this man-like attitude.”*

Rita’s circumstances mirror those of other women, such as Gabriela, a 35-year-old indigenous mother, and Ana, a 27-year-old mestiza, who experienced economic vulnerability in rural settings. Their experiences of IPV are exacerbated by intersecting challenges including racialized identities, societal status, and limited economic resources, particularly during the heightened IPV incidents amid the pandemic.

This situation contrasts sharply with women who possess greater social and economic resources and reside in urban and peri-urban areas. One example is Aranza, a 44-year-old dual U.S.–Mexican citizen, college-educated and a mother of four residing in an affluent neighborhood of Mexico City, who navigated her circumstances with the aid of her privileged social status. Her economic independence and social connections enabled her to access a variety of support networks. 

Aranza’s IPV experience worsened due to her ex-husband’s altered professional life. A prominent obstetrician reassigned from his usual duties, he then spent more time at home than before. This break in routine and loss of control escalated his abuse towards Aranza.

*“He would entertain himself here at home by constantly taunting me, claiming I had mental issues and a brain tumor, using his authority as a doctor. His subtle violence, coupled with my knowledge of his infidelities and lies, deeply affected me. He controlled and insulted me, calling me the worst wife, mother, and woman. But I knew I was smart and educated. I researched tumor symptoms and realized I did not have one… He was trying to gaslight me, but I recognized it.”*

Upon deciding to separate, Aranza managed a safe separation, supported by her financial autonomy and the ability to seek legal counsel. Her economic independence and social capital also enabled her to launch a self-funded social media advocacy movement with political impact.

*“I lived this process very alone initially during the second wave of the pandemic… I was isolated, filled with anguish… especially when access to my children was denied. The courts operating at reduced capacity slowed my fight for my rights even further… But then friends and other women I had connected with through social media and who were living these realities too, became a support network. We gathered at my house, subbed ‘the cuartel’ because this is where it all started, finding out that what we were living was violence, supporting each other, coming up with a plan, initiating a movement.”*

Despite facing numerous obstacles in seeking justice, her socio-economic influence ensured her complaints of family violence were taken seriously by the justice system. Discussing her interactions with the prosecutor’s office, she elaborated:

*“When I first approached the prosecutor’s office with my lawyer, the floor manager warned about the trouble I was inviting by pursuing justice against my ex’s violence, suggesting I didn’t understand who I was challenging… Now they don’t treat me that way, I have a strong lawyer… They know who I am [speaking to the legal backing she has garnered], they know what I represent, and we are nearing success in holding him accountable … But it has taken me years of time and money. I mean, seeking justice in this country is a full-time job.”*

Aranza’s social and cultural capital [36] equipped her with the necessary economic and political means to exit a violent relationship during confinement and navigate a partially operational patriarchal judicial system, albeit with challenges. Yet, her standing in an affluent circle also exposed her to unique forms of violence. Her partner, exploited his economic and social power, and the pandemic-induced judicial shutdowns, to commit vicarious violence, a particularly insidious form of abuse. He manipulated legal systems through actions like bribing judges, leveling baseless family violence charges against her, skewing legal processes to negate accusations against himself, and employing legal maneuvers to remove their children from their family home, denying her access to them indefinitely. The pandemic exacerbated Aranza’s challenges, leading to halts and delays in judicial proceedings. Consequently, she has faced prolonged legal battles and extended periods, stretching from months to years, where she has been unable to see her children.

*“It’s all about greed, making money off our suffering. It needs a lawyer with no morals to make a false accusation. This has become a modus operandi among prosecutors, lawyers, and judges, and the pandemic was the perfect time to carry all of this out. In our macho, violent country, these men know the system. They twist it to wipe you out of your children’s lives. They mix up criminal and family matters to legally take away your kids. And the institutions… buy into narratives like ‘she’s a bad woman, a bad mom, a bad citizen.’ So, the family court decides the kids should stay with a dad who was never there, never cared, and was the one causing harm from the start.”*

Aranza’s experience illuminates the nuanced interplay of privilege and IPV. On the one hand, her economic and social standing provided her with certain advantages in addressing IPV. This was particularly pertinent during the COVID-19 pandemic as she faced increased abuse. On the other hand, Aranza’s privileged position made her vulnerable to more subtle and hidden forms of abuse further exacerbated by pandemic-related disruptions, such as court closures and delays. Furthermore, her case underscores how affluence and influence in the pandemic environment could be exploited to impede legal actions and obstruct justice. Aranza’s heightened challenges of IPV during the pandemic is mirrored by the stories of Laura, Julia, and Beatriz, all women of middle-upper socio-economic status, with economic capacity and independence, and living in urban and peri-urban settings. 

Rita and Aranza, both encountered exacerbated forms of abuse during the pandemic, influenced by distinct factors. Rita’s pandemic-induced job loss eroded her financial independence, hindering her ability to leave her abuser and return to her rural hometown. Concurrently, her efforts to seek justice after increased violence were obstructed by the prosecutor’s office’s diminished operations and discrimination based on her identity and socio-economic status. Ultimately, her escape from cohabitation with her abuser was not a result of her own actions, but occurred unexpectedly when he disappeared one day. 

In contrast, the abuse Aranza faced intensified as her then partner spent more time at home. Nevertheless, her financial resources, support network, and connections enabled her to eventually escape the abuse and separate from her partner. Yet, her situation was complicated by her abuser’s social and economic capital [36], leading to vicarious violence. The pandemic’s strain on institutional capacities further diminished her avenues for support and intervention with this situation.

#### 5.2.2. Women’s Proximity to Abusers’ Support Network 

In our study, approximately half of the participants, including Luz, a 34-year-old indigenous woman of lower socio-economic status from rural Oaxaca, reported that residing near the families of their aggressors intensified their experiences of IPV during COVID-19, in contrast to other studies [37]. Luz lived in close quarters with her partner’s family, comprising his parents, his sister, and her children.

“*They were traditional, the men, typical machistas, the mother lived immersed in machista culture as well*”, she said. Luz referred to the attitude many women adopt in her community, where, with little awareness, they perpetuate gender inequalities, act submissively, serve the men in their surroundings and expect the same from other women. Despite recognizing the oppressive nature of these expectations, Luz described her marriage prior to the pandemic as *‘functional’*:

*“He used to be on the road for days as a truck driver, so I hardly knew his whereabouts. It was okay, not seeing him much around the house. Whenever he returned, it was always late, and he was beat. I’d do the usual wife stuff—cook, serve, then off to bed. We barely talked. I was kind of indifferent about him.”*

Prior to the pandemic, Luz recognized some forms of abuse in her life, but confinement during the pandemic dramatically worsened her household’s dynamics. The community’s lockdown limited entry and exit from the town, with tight security checks. All public facilities, businesses, and schools shut down. “*Suddenly, he was always there*”, Luz recounted. She noted that his income loss and limited movement led to more frequent and severe violence.

Luz described her partner’s aggression to be worse when he was drunk and “*during those months he was getting drunk inside of the house and things got more tense*” said Luz, implying a rise of alcohol-induced aggression because of her partner’s forced homestay due to the pandemic.

The psychological abuse, on the other hand, was insidiously pervasive and harder to recognize at the time. “*I was always told I was crazy*”, she shared, recounting how the family discouraged her from questioning her husband’s behavior. “*It kept me there, silent, many times… but during the pandemic his presence really started to bother me, and I started to realize things about his infidelities and his finances*”, she shared.

This escalation in domestic tension reached a critical point during the first 6 months of confinement, coinciding with her husband spending more time at home. In delineating this crucial moment, Luz shed light on the family’s complicit role in perpetuating the violence: “*The family would just look the other way when he was violent*” she explained, “*I had normalized a lot of it … but after a particular instance where he threw something at my son and then crushed my finger with the door, I decided to actively involve his parents*”.

Upon confiding in her in-laws, her father-in-law dismissed her concerns, while her mother-in-law responded by pressuring her. “*She would say it was my duty to stay, that I had married her son and that I could not leave him*”, Luz recounted. Despite Luz’s attempts to dissolve their relationship, physical separation was unattainable due to the community’s confinement measures. Moreover, she and her children lacked alternative housing options due to financial constraints. Additionally, her husband’s family exerted constant pressure on her to stay, making it an emotionally taxing time. Luz’s struggle to separate from her violent partner illustrates the emotionally complex task of addressing IPV within a shared extended family setting. The COVID-19 pandemic’s isolation measures exacerbated these difficulties, trapping women with their abuser’s network, thereby increasing their vulnerability. This proximity during the pandemic not only intensified IPV experiences but also obstructed escape attempts.

Luz’s situation contrasts sharply with urban women’s experiences, who typically were able to physically separate from their partners and live farther from the abuser’s family, without the stringent mobility restrictions imposed in rural areas. In our study, this urban group mainly includes women from middle-class and higher socio-economic backgrounds, benefiting from greater financial independence and familial support.

Laura, a 43-year-old mestiza and mother of two living in an upscale neighborhood in Mexico City, recounts her concealed struggle with IPV. Married to a businessman deeply invested in his public image, Laura’s life was eclipsed by his reputation obsession, cloaking her IPV experiences behind a veneer of upper-middle-class familial harmony. The COVID-19 pandemic worsened these conditions, as lockdowns increased the violence’s severity, and added pressures like homeschooling strained their household further. When her usual support network became inaccessible, Laura moved to her mother’s home early in the pandemic, a key step towards her safety and autonomy. Despite some social pressures to preserve her marriage, she found considerable support from her friends. This contrasted with her ex-partner’s network which remained oblivious to the situation:

*“When he was with our social circle you wouldn’t even imagine he was the same person, he was the most enchanting, the most helpful, the most sweet and caring. My family thought he was the most wonderful thing to ever happen to this world. But my mom supported me in my decision to leave… I think his family believed him; he is an expert liar.”*

After months of escalating aggression from her partner, worsened by more time spent at home during the pandemic, a particularly intense verbal altercation happened.

This incident, more severe than previous ones, led Laura to fear a physical confrontation and call the police. She managed to leave without facing external opposition, supported by her community and family. 

Similarly, Beatriz and Julia, living in and near Mexico City, faced IPV without being physically close to their aggressors’ networks or confined due to COVID-19-induced measures. Their abusers, notable for their political and professional influence, primarily inflicted violence in private, often without witnesses. In their cases, financial stability and autonomous living arrangements were crucial in breaking free from violent partnerships, especially in conditions marked by isolation, restricted mobility, and increased contact with the aggressor. The location of their residences plays a significant role in mitigating the exacerbating effects of COVID-19 measures. Although navigating these challenges is emotionally taxing, the women’s specific circumstances helped them initially manage and eventually escape violent situations, even during the height of the COVID-19 crisis. A notable advantage was the limited involvement of the abuser’s support network.

It is clear that understanding the relationship between the pandemic, confinement, and IPV across different living environments requires examining diverse factors such as socio-economic status, co-residence with the aggressor, proximity to their network, and physical mobility.

### 5.3. The Paradoxical Effects of COVID-19 Measures on IPV for Women

This study confirms that IPV in Mexico intensified during the COVID-19 pandemic, consistent with previous findings, but also presents unexpected results. In our sample, women from rural or urban fringe areas with middle to lower economic backgrounds saw a decrease in IPV in some instances, attributed to pandemic-induced social distancing and isolation policies. Specifically, mobility restrictions in rural areas curtailed access to these communities, reducing encounters with abusers for those not living together. Furthermore, the reduction of social gatherings, especially those involving alcohol, led to fewer incidents of alcohol-related violence. These outcomes, though seemingly contradictory amid rising IPV, underscore the intricate effects of public health measures across different communities, locations, and support systems.

#### 5.3.1. Restrictions on Geographical Mobility as a Protective Factor against IPV

Before the pandemic, Isabel experienced severe violence in her relationship and subsequently left her partner’s community. Moving to a peri-urban municipality in the peripheries of Oaxaca City, she achieved a degree of independence, supporting herself and her children with a teaching job. Yet, she faced a different IPV pattern: her partner withdrew financial support and engaged in digital harassment, exacerbating harm and preventing her from fully recovering from her trauma [38]. This reflects Walker’s Cycle of Abuse theory [39], showing a cyclical pattern of abuse, separation, and reconciliation. Isabel’s cycle included violent episodes, physical separation, and periods of reconciliation influenced by emotional manipulation and co-parenting desires, often leading back to cohabitation and continuing the cycle of abuse.

Despite a lengthy period of physical separation immediately previous to the pandemic, she felt their relationship was “*in repair mode*” and improving, especially following the birth of their second child, Samuel. “*About 15 days earlier* [prior to the birth], *they declared the pandemic and the self-isolation recommendations*”, she recounted. At 40 days postpartum, Isabel planned to join her partner in his rural community to avoid COVID-19’s urban impact, “*so that I wouldn’t be alone here with the children and so that we can take shelter from COVID-19, because in the rural communities it didn’t hit as hard as in the city.”* she explained. Unlike the urban cities and the sub-urban peripheries, many rural communities like that of her ex-partner isolated themselves [40]: “*Just when we planned for me to return to the community, they closed the passage. The rural communities became completely hermetic. So I could no longer enter his community, nor could he leave it,*” Isabel explained. This blockade prevented her from re-entering his community, effectively interrupting a recurring cycle of IPV.

Isabel described how this change of plans resulted in escalating psychological attacks and digital harassment, leading to her blocking contact with him, this time without fear that he could come looking for her. It was during this period of safe distance, brought on by COVID-induced geographical immobility, that Isabel experienced a profound paradigm shift. The enforced physical distance and cessation of communication with her abuser instilled in her a newfound sense of safety, agency, and capability. She highlighted the critical role of enforced separation in fostering her autonomy and resilience:

*“My emotional stability depended on being physically alone… where I had the opportunity to analyze myself. And don’t think that it was easy, there have been very sad days when I have cried, felt very tired, when I don’t want to be a mom anymore, or where I want someone to come and help me. But this part of sharing alone with my children has helped me so much.”*

Isabel’s narrative sheds light on the intricate dynamics of IPV, illustrating the pivotal role of enforced physical distancing during the COVID-19 pandemic in facilitating her separation from her abuser. While geographical separation alone was not the sole factor in Isabel’s disengagement, it was instrumental. The mix of COVID-19 community preventive measures—enhancing her sense of autonomy and providing space for reflective healing, along with sustained distance—were key in breaking free from the abusive relationship and starting her healing process.

Ultimately, COVID-19 measures provided Isabel with an opportunity for prolonged physical and emotional distance from her abuser, a decision initially imposed by circumstances beyond her control. This unintended intervention allowed her to slowly claim autonomy, shielded from her ex-partner’s immediate retaliation. Her story highlights the complex interaction between personal agency and external influences in escaping IPV, emphasizing the critical need for support systems that empower those in similar situations.

#### 5.3.2. Decrease in Access to Alcohol

Jessica, a 37-year-old indigenous mother of two from a rural area near Oaxaca City, linked her experience of IPV to the widespread problem of alcohol abuse in her community. Her husband, who would socially binge drink, exemplifies how alcohol use in social contexts heightens aggression, contributing to DV. His weekly drinking episodes resulted in sexual and physical abuse towards Jessica. She recalled a particularly harrowing incident just a year before the pandemic, shortly after the birth of her second child. “*About 5 days after giving birth, he came home drunk… ranting about many things.*” Her husband’s drunken tirade accused her of ruining his life, a claim that bewildered Jessica, given she had just delivered their son. “*It cannot be me he’s talking about... I just gave birth,*” she said, her voice cracking. He then physically assaulted her, even pulling at her cesarean section bandages. Despite her wish to leave, her postpartum vulnerability and the newborn’s needs made it impossible. “*I didn’t have the strength to leave him then,*” she confessed.

This incident led them to couples therapy where Jessica confronted his drinking: “*I asked him to cut back... ‘I don’t mind if you drink, but don’t overdo it,*’” she urged. However, the therapy saw no real improvement, as his abusive episodes persisted and intensified.

“*Then, the pandemic struck*”, Jessica reflected. She noted an initial shift in dynamics: “*At first, things seemed to change.”* In this period, despite the federal government’s recommendation for voluntary confinement and self-isolation, her community enacted a complete halt of work and social activities.

Jessica and her partner, proprietors of a photography business reliant on social gatherings, were significantly impacted by the economic downturn. While the immediate financial strain was evident, the broader, less visible effects of the town’s lockdown in those initial months were multifaceted. In Jessica’s case, the enforced social isolation during the pandemic played a crucial role in reducing IPV incidents by limiting her husband’s social activities and, consequently, his alcohol consumption. This absence of alcohol, typically a precursor to his violence, led to a period of relative calm. “*We got along okay*”, she noted, “*there were no gatherings with friends or family, so no alcohol, and, as a result, no violent episodes for a while.*”

In Jessica’s community, while there was no formal alcohol ban, the pandemic’s social restrictions prevented habitual social drinkers from engaging in their usual patterns of behavior, leading to less aggression. This suggests that aggression is often exacerbated by alcohol and highlights how external measures can effectively mitigate aggressive triggers.

## 6. Discussion

### 6.1. IPV before the Pandemic

Interviews with our participants revealed a consistent IPV pattern that supports prior research [23,41,42]: women from all socio-economic backgrounds experienced some form of IPV prior to the COVID-19 pandemic.

The nature and severity of IPV prior to the pandemic also varied significantly across socio-economic strata, a situation that we found persisted for IPV after the onset of the pandemic. Women from lower socio-economic backgrounds experienced higher incidents of physical and sexual violence, often in front of bystanders, signaling a disregard by their aggressors for public image. Their economic dependence on partners was marked by restrictive control over finances, including the denial of access to funds. This compounded vulnerability, intensified by societal pressures and ostracization, drastically limited their ability to seek and access supportive networks.

Women from upper-middle and upper socio-economic backgrounds reported fewer instances of physical and sexual violence, with just one case of physical violence noted. These women enjoyed greater economic independence through personal income or family wealth, which shifted the nature of economic violence they faced towards exploitation tactics such as deceit and manipulation for financial gain. Their aggressors, prioritizing their public image, often kept their abusive behaviors private. Additionally, these women received more support, validation, and protection from their social networks.

These findings align with previous research showing that before the pandemic, around 35% of women globally experienced IPV [42]. They are also supported by other studies that report risk factors for IPV, including lower socio-economic status, unemployment or financial dependence on the perpetrator, limited social support, geographic isolation, and community tolerance of IPV [23,41,42]. A correlation was noted between middle and lower socio-economic status and IPV incidence before the pandemic [42], but, unlike our study, no distinctions were made between the types of violence women were facing. Our findings concur with some studies that conclude that women are generally more subjected to psychological violence than to other forms of IPV [42], with racialized and indigenous women facing higher IPV rates in general [41].

### 6.2. The Negative Influence of the Pandemic and COVID-Induced Measures

Consistent with past studies [42,43], our research found that IPV increased for all women during the pandemic. However, the increase was not uniform. The escalation varied significantly at the interplay between women’s socio-economic status, rural vs. urban residence, and the proximity or distance to abusers’ support networks.

*Women of lower socio-economic status* faced increased challenges due to pandemic-related job losses, which eroded their financial independence and restricted their ability to leave abusers. Municipal restrictions in rural areas prevented return to family homes and escape from abusers’ communities, and efforts to seek justice were hampered by reduced operations in prosecutor’s offices and racial and ethnic discrimination. Escape from abusers depended on external factors rather than individual actions.

Conversely, women from *upper socio-economic backgrounds* encountered increased abuse due to partners’ stress from pandemic-induced workplace changes and more time spent at home. However, their financial independence, support networks, and connections facilitated their escape from abusive situations. Yet, the abusers’ high socio-economic standing complicated the abuse beyond the home, leading to post-separation judicially permissive vicarious violence. The pandemic also strained institutional support and intervention capacities, affecting these women’s access to help; however, this was not racially or ethnically based.

We found that approximately 50% of participants reported an intensification of IPV during the COVID-19 pandemic in part attributed to living in close proximity to the families of their aggressors. This group consisted exclusively of women of lower socio-economic status and the majority from rural Oaxaca where traditional gender roles and economic imperatives frequently compelled women to relocate near or within their partner’s family residence. This relocation embedded them within their partner’s familial support network, as other studies highlight [16]. Extensive research has examined the dynamics of IPV in rural contexts [16,44,45,46]. Women cohabiting with their abuser’s extended family not only endure violence from their partner but also face the complicity of bystanders [47]— the family members and community members who often witness, fail to intervene, and normalize such acts. In our study, the situation was further aggravated by municipality-imposed COVID-19-related mobility restrictions and increased home confinement—policy measures that were common in rural Mexico [40]—leading to a marked negative impact on the women’s ability to escape IPV.

Conversely, women residing in urban and peri-urban settings predominantly from middle-class and higher socio-economic status, reported living independently, away from their abuser’s family, often in areas that were either neutral or closer to their own support networks, and not subject to the same degree of COVID-19 mobility restrictions as those in rural areas. These participants indicated that their abusers typically perpetrated violence in private, without witnesses. The geographical and social distance from the abuser’s family and network, coupled with closer proximity to their own support systems, significantly mitigated the adverse effects of COVID-19 measures on IPV. This separation facilitated their initial management and eventual resolution of violent situations, even at the height of the COVID-19 crisis. This finding aligns with previous research by Peled and Krigel [48] on the importance of financial stability and autonomous living arrangements to mitigate the experience of IPV.

### 6.3. The Paradoxical Effects of COVID-19 Measures on IPV for Women

In our study, women from rural areas or urban peripheries, predominantly of middle to lower socio-economic status, paradoxically experienced certain benefits from the social distancing and isolation policies enacted by local authorities during the pandemic.

Specifically, these policies contributed to a momentary or, in a few cases, a permanent decrease in IPV in two main ways. Firstly, the enforcement of geographical mobility restrictions by local authorities resulted in a marked reduction in contact with abusers for those women who were not cohabitating with their partners. This enforced separation provided them with an opportunity to enhance their autonomy, engage in reflection and healing, and maintain their physical and emotional safety, thereby interrupting cycles of abuse and initiating a process of recovery.

Secondly, the pandemic led to a reduction in social gatherings, which women report often involved heavy drinking. The restrictions on social activities disrupted habitual patterns of social drinking, subsequently reducing alcohol-fueled aggression within households. Studies like Silverio-Murillo et al. [49] indicate that reduced alcohol consumption during the pandemic had little effect on DV in urban areas like Mexico City. However, in more peripheral or rural areas with stricter isolation measures, the outcomes might diverge, as our study suggests. This aligns with research indicating that obstacles to alcohol access, as observed by Markowitz [50], correlate with a significant decrease in IPV, including in sexual assault [5]. Furthermore, broader studies indicate that lowering alcohol consumption can decrease overall crime rates. Specifically, alcohol sales bans during the pandemic have been linked to reduced violent crime in certain areas [5].

These findings suggest a more complex and nuanced relationship between the pandemic’s progression and the dynamics of IPV, at least in relation to social distancing and confinement, as well as alcohol consumption by abusers.

## 7. Limitations and Future Research

Our study offers valuable insights into the prevalence and patterns of IPV during the COVID-19 pandemic in Mexico, yet it is not without its limitations.

### 7.1. Participant Selection

One primary constraint is the sample size; with only 10 ethnographic interviews, our research provides in-depth qualitative data but limits the ability to generalize these findings across the broader population. The depth of qualitative research lies in its detailed narrative, but a larger sample would provide a more representative view of the diverse experiences of Mexican women.

Another significant limitation is the demographic scope of our study. Notably, our research did not include Afro Mexican women, trans women, or women from the northern regions of Mexico. Given IPV’s socio-demographic correlations with ethnic and racial identities, as well as socio-economic status, the exclusion of these groups presents a gap in our understanding of the full spectrum of IPV experiences during the pandemic.

To build upon the findings of this study, future research should aim to expand both in scale and participant diversity. A larger scale study that integrates a quantitative approach would complement the rich qualitative insights gained here. Quantitative methods would enable the identification of broader trends and correlations, enhancing our understanding of IPV during pandemic conditions. Future studies should prioritize inclusivity in participant selection, especially including Afro Mexican women, trans women, and women from northern Mexico. This would ensure a more comprehensive understanding of IPV across different socio-cultural and regional backgrounds.

### 7.2. Controlling for Influential Variables

Future research should control for various factors that may impact experiences of IPV. These include socio-economic status, ethnic and racial background, rural versus urban living conditions, and the presence or absence of family and friend networks. Such an approach would allow for a deeper analysis of how these variables influence IPV patterns during crises like the COVID-19 pandemic.

### 7.3. Subject of Study

An intriguing direction for future research involves a deeper examination of the contradictory outcomes associated with interventions implemented to mitigate COVID-19 on IPV. Our findings suggest that pandemic-imposed restrictions, notably the curtailment of geographical mobility and the diminished availability of alcohol, provided some protective mechanisms for women experiencing IPV. A more thorough exploration of these and other restriction elements could yield critical insights relevant to the formulation of public policy and the development of crisis management strategies. Additionally, comparative analyses of the effectiveness of municipal governments versus federal directives in enacting public health policies and interventions merit further study. Such research is particularly pertinent for enhancing our understanding of effective measures at both the local, state and national levels, especially in the context of addressing the public health crisis of gender-based violence within the context of Mexico.

## 8. Conclusions

This study presents an analysis of IPV in Mexico, accentuated under the intensified strain of the COVID-19 pandemic and the federal-, state-, and municipal-level measures taken to mitigate its effects. It highlights pre-pandemic high IPV levels, noting different experiences based on socio-economic status, with poorer women experiencing higher rates of physical and sexual violence, economic control, and societal ostracization, and lower levels of bystander intervention or access to institutional support.

Our research uncovers the complex ways COVID-19 exacerbated IPV and existing inequalities. IPV during the pandemic disproportionately affected women with marginalized identities, limited economic means, reduced support networks, and residing in remote areas in close proximity to their abuser’s networks. Women of lower socio-economic status, especially in rural areas, faced heightened challenges due to financial dependencies on abusers, cohabitation or close proximity to abuser’s networks, and mobility restrictions due to municipal-level COVID-19 lockdowns. In contrast, wealthier women encountered increased psychological and economic abuse from partners in confinement but had more resources and greater economic and geographical mobility to escape IPV. This study also highlights the emergence of pandemic measures as protective factors for IPV, offering some women a reduced contact with abusers and a break from patterns of social drinking that fueled aggression.

Insights into these aspects were attainable through in-depth interviews and participant observation, underscoring the importance of qualitative methods in uncovering nuanced effects of and hidden patterns in IPV dynamics. This approach is universally applicable, emphasizing the need for qualitative research in the exploration of IPV.

The study sheds light on IPV as a universal problem that transcends geographical and socio-cultural boundaries. The global context of the pandemic provides a backdrop against which the specifics of IPV in Mexico offer insights into similar patterns and differences worldwide. This focus on marginalized identities, economic limitations, and remote living conditions is highly relevant internationally, guiding global efforts toward more inclusive and effective support structures. Furthermore, the nuanced understanding of pandemic confinement measures’ mixed effects, acting as both risk and protective factors, offers critical knowledge for managing public health crises while preventing IPV. The confrontation of stereotypes around socio-economic status challenges misconceptions and fosters a nuanced global discourse on IPV.

Our findings illuminate the complex interplay of identity, individual agency, stress, caring for and protecting children, and external factors in coping with and navigating away from IPV, underscoring the urgent need for adaptable and resilient support systems. This necessitates a comprehensive re-evaluation of existing IPV response mechanisms, considering multifaceted factors that shape women’s experiences of violence. Such an understanding is pivotal for developing holistic strategies that address the physical, psychological, and economic challenges of escaping and recovering from abusive relationships globally. The insights from this study are not only significant for the Mexican context but also hold value for the international scientific community and global efforts to combat IPV. The findings underscore the importance of tailoring support systems and emergency responses to the realities of individuals in diverse situations, aiming not only for immediate physical safety but also for fostering an environment that supports long-term emotional recovery and resilience.

## Figures and Tables

**Table 1:** Participant’s Demographics.

Name	Ethnicity	Age	Socio- Economic Status	Economic Dependence	Education	Residence	Location	COVID-19-Measures Authoritative Body of Government
Isabel	Indigenous	35	Middle socio- economic status	Independent	Preparatory school & teaching certificate	Peri-urban	State of Oaxaca	Municipal
Rita	Indigenous	42	Low socio- economic status	Independent	Primary School	Urban	State of Oaxaca	Federal
Beatriz	Mestiza	41	Upper-middle socio- economic status	Independent	Masters	Peri-urban	State of Mexico	Federal
Julia	White Mexican	40	Upper socio-economic status	Independent	Undergraduate University	Peri-urban	State of Mexico	Federal
Aranza	White Mexican	43	Upper socio-economic status	Dependent	Undergraduate University	Urban	Mexico City	Federal
Luz	Indigenous	34	Low socio-economic status	Dependent	Secondary School	Rural	State of Oaxaca	Municipal
Laura	Mestiza	47	Upper-middle socio-economic status	Independent	Undergraduate Degree	Urban	Mexico City	Federal
Jessica	Mestiza	37	Middle socio-economic status	Independent	Preparatory School & teaching certificate	Peri-urban	State of Oaxaca	Municipal
Gabriela	Indigenous	33	Low socio economic status	Dependent	Undergraduate University & counseling certificate	Rural	State of Oaxaca	Municipal
Ana	Mestiza	32	Low socio-economic status	Dependent	Preparatory School	Rural	State of Oaxaca	Municipal

## Data Availability

The datasets presented in this article are not readily available because they are derived from extensive qualitative interviews to which women did not provide explicit consent for their full disclosure, thereby restricting their availability for public access. Requests to access quotes from the datasets should be directed to sofia.navarrete-zur@fulbrightmail.org.
